# Challenges of Interstitial Ectopic Pregnancy in a Fibroid Uterus: A Case Report

**DOI:** 10.1155/crog/5737675

**Published:** 2026-05-13

**Authors:** Orla Power, Joshil Selvajothi, Louise Dooley, Tamara Kalisse, Deirdre Hayes-Ryan

**Affiliations:** ^1^ Department of Obstetrics and Gynaecology, Cork University Maternity Hospital, Cork, Ireland, ucc.ie; ^2^ Pregnancy Loss Research Group, University College Cork, Cork, Ireland, ucc.ie; ^3^ National Perinatal Epidemiology Centre, University College Cork, Cork, Ireland, ucc.ie

## Abstract

**Introduction:**

Interstitial ectopic pregnancy (IEP), although a rare clinical entity representing 2%–6% of all ectopic pregnancies, is associated with higher morbidity and mortality than other types of ectopic pregnancy, compounded by distinct challenges in diagnosis and management.

**Case Presentation:**

A 40‐year‐old multiparous female (G7P3M2E1) presented for routine early pregnancy ultrasound due to a previous history of pregnancy of unknown location (presumed tubal ectopic pregnancy). Transvaginal ultrasound scan did not identify any intrauterine gestational sac, despite *β*‐hCG levels elevated at 19,009 iU/L. Imaging was noted to be technically challenging due to the presence of a large fundal fibroid. Serial measurement noted a drop in *β*‐hCG levels and the patient was initially managed conservatively as a pregnancy of unknown location. After becoming acutely unwell, ruptured ectopic pregnancy was the primary differential diagnosis. The patient underwent emergency laparoscopy, where a right IEP was diagnosed with associated large haemoperitoneum. Right superficial cornuostomy and right salpingectomy were performed, and the patient had an excellent recovery.

**Conclusion:**

Challenges in diagnosis and management of IEP are recognised; however, these are further complicated in situations where there is coexistence of other uterine pathology such as large uterine fibroids. This case highlights that with appropriate expertise, safe and successful surgical management of ruptured IEP is possible using a laparoscopic approach, with cornuostomy and ipsilateral salpingectomy.

## 1. Introduction

Interstitial ectopic pregnancy (IEP) occurs when the fertilised egg (blastocyst) implants in the most proximal (interstitial) portion of the fallopian tube [1]. This rare type of ectopic pregnancy is associated with unique diagnostic challenges, which in combination with the location of the gestation within the highly vascular myometrium, contribute to higher maternal morbidity and mortality, thought to be up to seven times greater than other ectopic pregnancy variants [2]. Expectant, medical, and surgical approaches are described in the management of IEP. We present a case of IEP occurring in a fibroid uterus, elucidate the associated challenges and demonstrate safe laparoscopic surgical management of IEP even in a complex scenario.

## 2. Case Presentation

**Figure 1:**
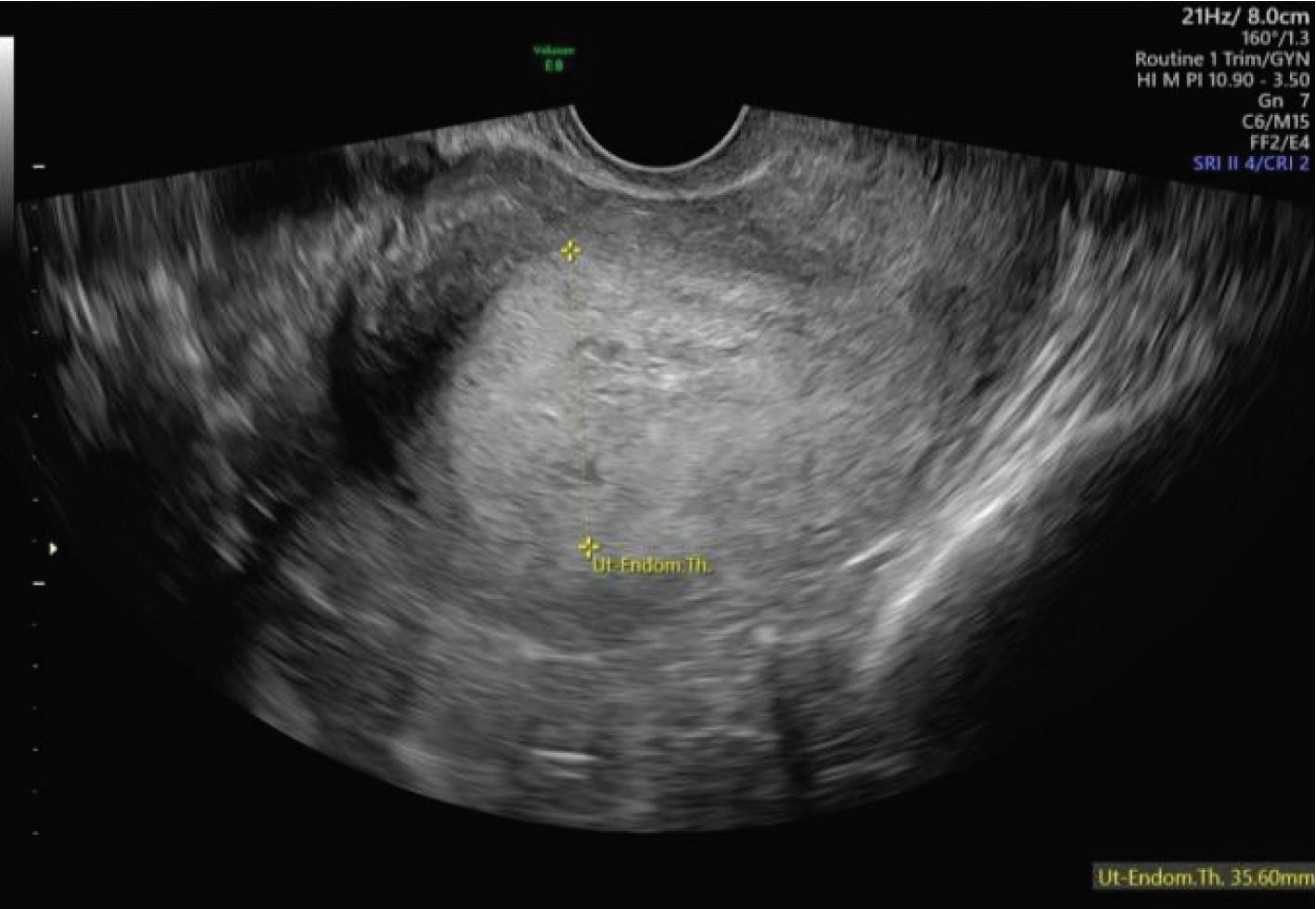
TVS image of thickened endometrium.

**Figure 2:**
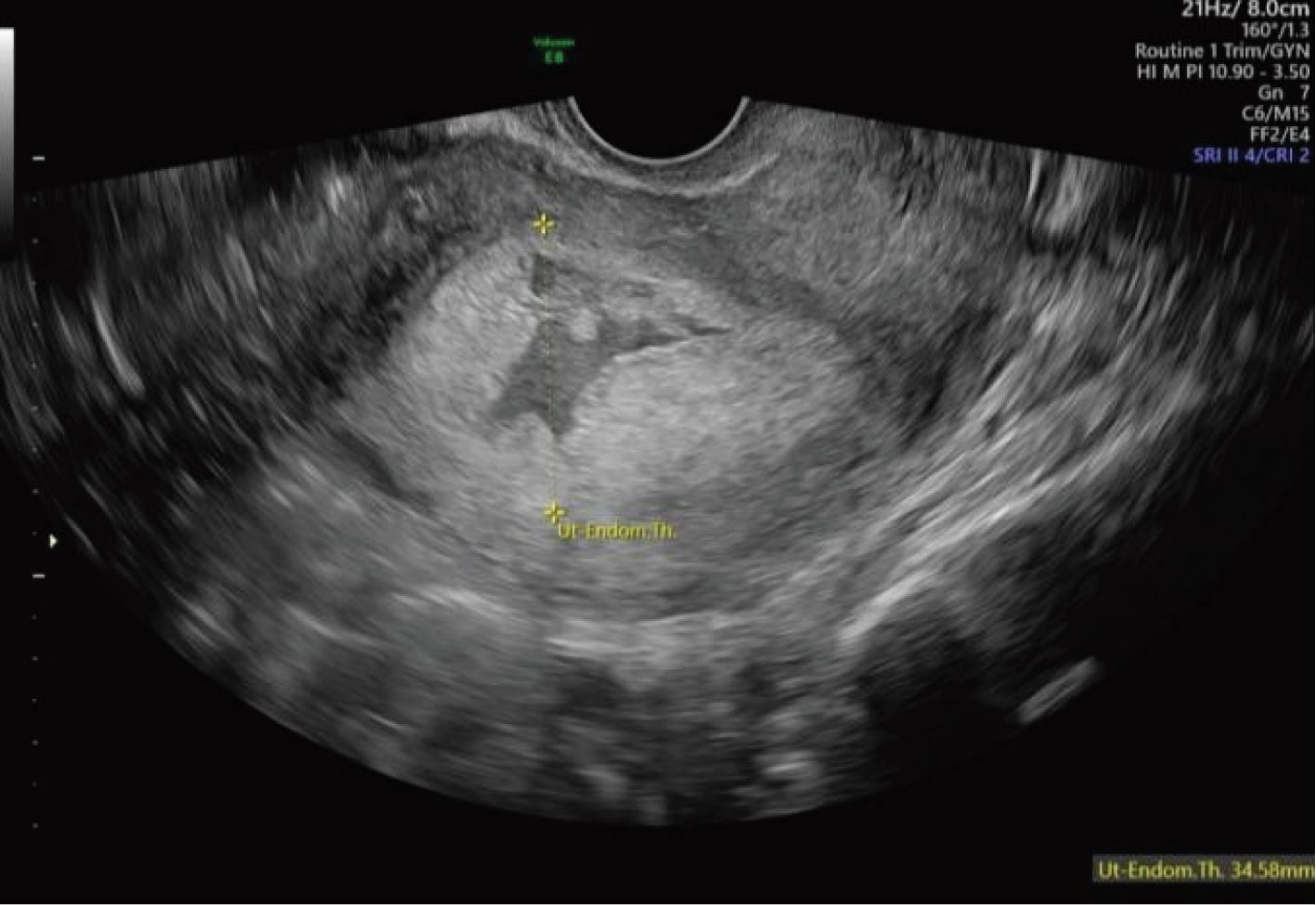
TVS image of hypoechoic area within endometrium.

**Figure 3:**
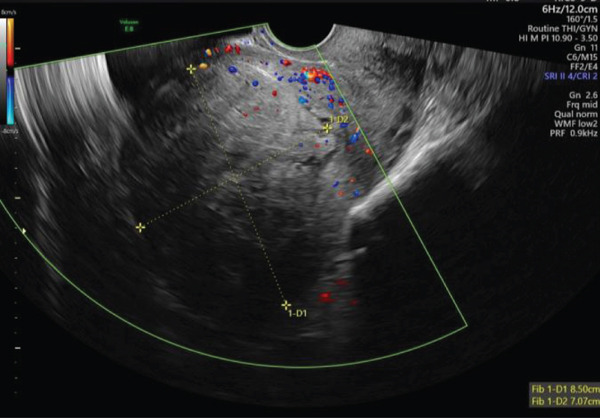
TVS image of fundal fibroid.

A 40‐year‐old female, gravida 7, para 3 + 3, was referred to the early pregnancy unit (EPU) of a large tertiary maternity hospital at 5 weeks′ gestation for a pregnancy localisation scan, due to a previous history of pregnancy of unknown location 6 years prior. Her background history included three previous spontaneous vaginal deliveries at term, two first trimester miscarriages and the previously mentioned pregnancy of unknown location for which she had successfully undergone medical management with methotrexate. On review in the EPU at a gestational age of 6 weeks and 2 days based on her last menstrual period, the patient reported feeling well, with no abdominal pain or vaginal bleeding. Transabdominal (TAS) and transvaginal ultrasound (TVS) revealed a thickened 35‐mm endometrium (Figure 1) containing a tiny hypoechoic area (Figure 2), but no clear intrauterine gestational sac (IUGS). Views were limited by the presence of a large fundal fibroid with a mean measurement of 80.2 mm (Figure 3). *β*‐hCG was significantly elevated at 19,009 iU/L. Given the high *β*‐hCG in the setting of a PUL with poor views due to a large fibroid, the patient was considered high risk of ectopic pregnancy. Diagnostic laparoscopy was considered but as she was clinically well, a decision was made to manage conservatively and a return appointment made for repeat ultrasound and *β*‐hCG in 48 h. She was counselled regarding warning signs of ectopic pregnancy rupture and advised to present immediately to the emergency department if any concerns.

During this second appointment, the patient reported mild discomfort in the right iliac fossa and suprapubic area, but no vaginal bleeding. On examination, the abdomen was soft and nontender, but some discomfort was noted on palpation of a bulky uterine fundus, presumed secondary to the large fundal fibroid. No cervical excitation was elicited on bimanual examination. *β*‐hCG levels showed a reduction to 14,222 iU/L; hence, conservative management was continued on an outpatient basis.

A third appointment was arranged for 72 h later, at which point a further reduction in *β*‐hCG to 7,517 iU/L was noted. The patient reported ongoing light vaginal bleeding, without pain. TVS by two experienced sonographers revealed a persistently thickened endometrium, 32.1 mm, with no pregnancy or pregnancy tissue identified within. Views of the uterus and right adnexa were noted to be technically difficult due to the presence of the previously noted fibroid, now measured at 76 × 74 mm with an area of degeneration noted within. The right ovary was thought to contain a probable corpus luteal cyst and was otherwise unremarkable. No free fluid was observed. Whilst awaiting medical review following this ultrasound scan, the patient became acutely unwell. She complained of severe lower abdominal pain, worst in the right iliac fossa.

**Figure 4:**
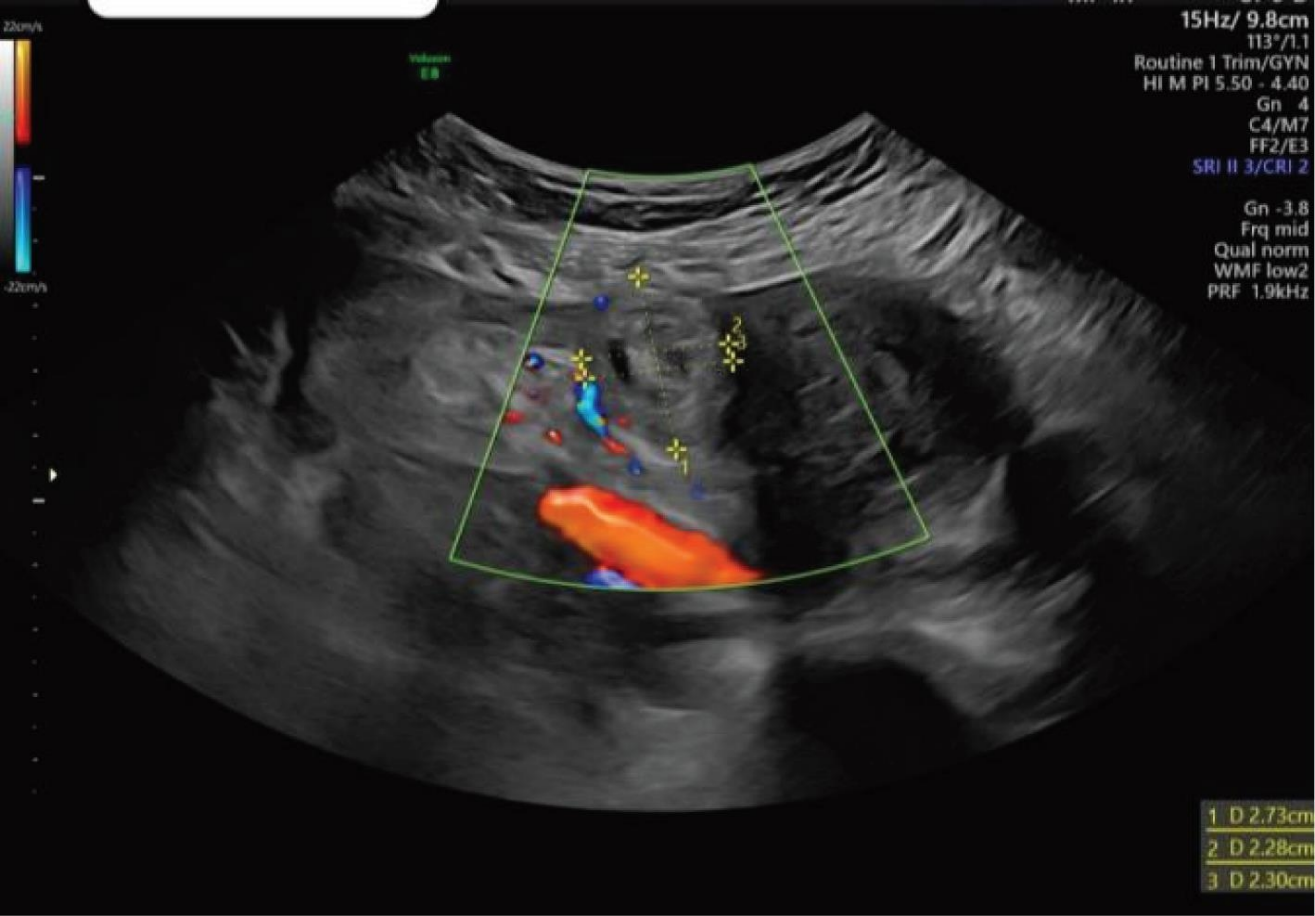
TAS image of suspected right IEP.

TAS and TVS were repeated with findings similar to that above 1 h previously. Of note, there was no evidence of free fluid; however, cervical excitation was now noted. TAS was suspicious for a right‐sided adnexal mass adjacent and separate to the right ovary, close to the uterus, measuring 2.7 × 2.8 cm, with colour flow noted when Doppler was applied. Views were described as very limited due to shadowing from the fibroid, but the clinical presentation and ultrasound findings were highly suspicious for ruptured right IEP (Figure 4). Differential diagnosis included ruptured intramural ectopic pregnancy; however, given the location of the adnexal mass and lack of previous uterine surgery, this was considered unlikely.

**Figure 5:**
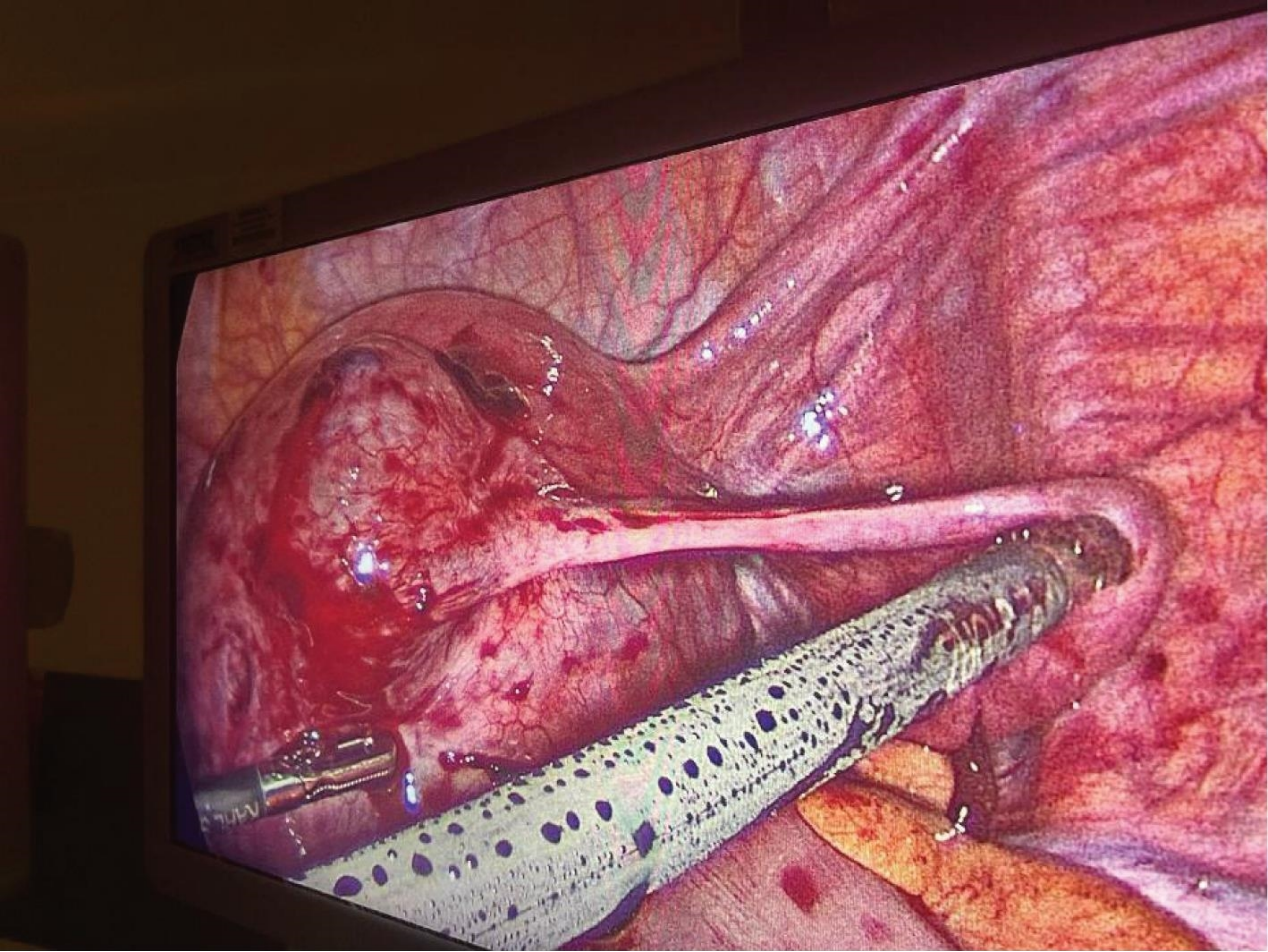
Diagnostic laparoscopy findings of a ruptured right‐sided IEP at the very distal end of the right fallopian tube surrounded by an overlying myometrium.

Emergency management was initiated including establishment of intravenous access and obtaining blood samples for group and cross‐match, initial fluid resuscitation instigated, and the patient was transferred urgently to the operating theatre for surgical management in the form of laparoscopy. Following laparoscopic entry using a direct entry port, a probable right IEP measuring 2 × 2 cm was observed. A superficial rupture with an identifiable bleeding point was evident, with associated haemoperitoneum of 1 L. Right IEP was confirmed clinically with the ruptured ectopic pregnancy observed to be involving the right fallopian tube at its proximal portion where it traversed the myometrium. Histology subsequently confirmed the pregnancy implantation site as the proximal portion of the fallopian tube. The left fallopian tube appeared normal, as did the ovaries bilaterally. The uterus was noted to be bulky due to the presence of a large intramural fibroid (Figure 5).

**Figure 6:**
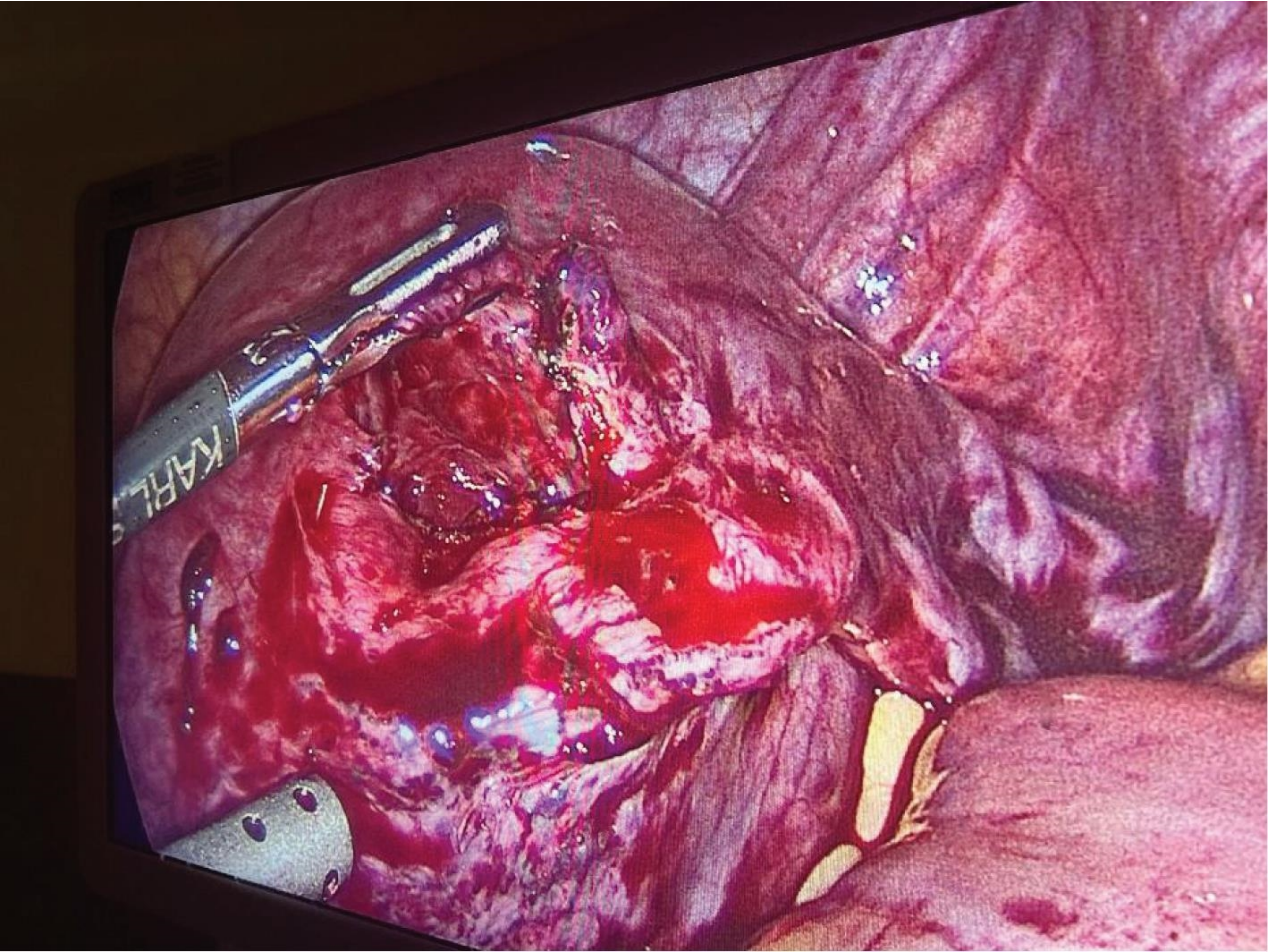
Post removal of the IEP by right cornuostomy, prior to defect repair and right salpingectomy.

The surgical team proceeded with laparoscopic right superficial cornuostomy and right salpingectomy. Vasopressin was injected around the ectopic site, followed by a linear incision of the gestational sac and aspiration of contents. Barbed continuous suture was utilised and haemostasis achieved without breaching the uterine cavity or removal of any uterine muscle. The gestational sac/pregnancy tissue was sent for histological examination. Following thorough suction and irrigation, the uterine serosa over the cornua was closed using 3.0 V‐lock suture. Right salpingectomy was then performed using a bipolar sealing device. After further suction and irrigation, haemostasis was confirmed (Figure 6).

**Figure 7:**
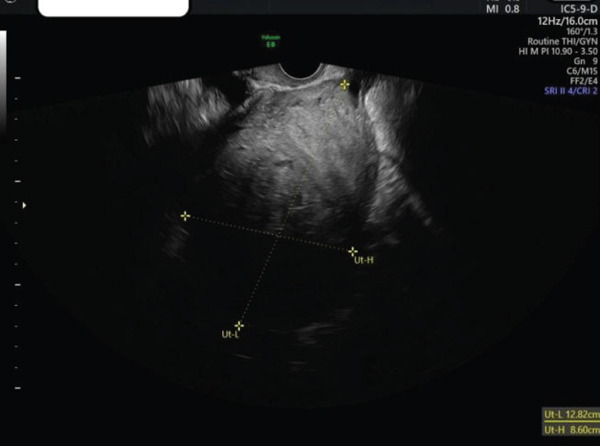
TVS image of fibroid post‐op.

**Figure 8:**
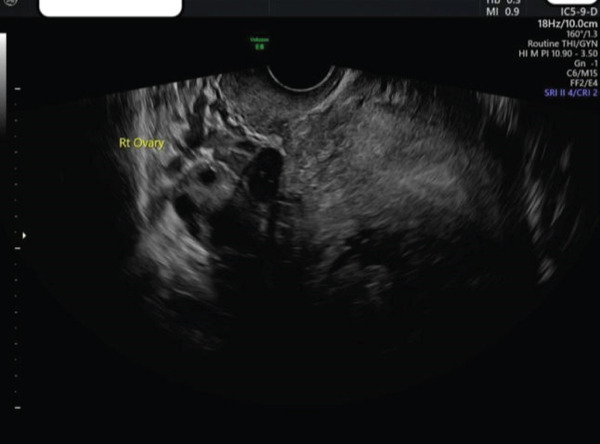
TVS image of right adnexa post‐op with poor views.

The patient made an excellent postoperative recovery and was discharged home the following day. She underwent weekly *β*‐hCG follow‐up until normal at 29 days post‐op. She returned for follow‐up ultrasound scan 6 weeks later, which confirmed the presence of a large fundal fibroid (Figure 7), with associated challenging views of the uterus, endometrium and adnexae on each of TAS and TVS (Figure 8).

## 3. Discussion

### 3.1. Diagnosis and Terminology

IEP is a rare type of ectopic pregnancy, defined as an ‘ectopic gestation implanting in the most proximal part of the fallopian tube’ [1]. Although accounting for just 2%–6% of all ectopic pregnancies, IEP is associated with unique diagnostic challenges and carries a high risk of maternal morbidity and mortality, largely related to delayed diagnosis, presentation at more advanced gestation, and implantation of the gestation in the highly vascular myometrium contributing to the risk of shock and haemoperitoneum with rupture. Incidence is observed to be increasing, which may be related to increasing use of assisted reproductive technology [3].

There has been much debate about nomenclature used to describe IEP, and there is an absence of a comprehensive classification system for ectopic pregnancies [4]. Recent recommendations in this regard have been developed by an ESHRE working group on the topic, in progress towards uniform guidelines fit for use in clinical practice [4].

Historically IEP terminology has been used interchangeably with both cornual ectopic pregnancy and angular pregnancy, but the latter terms are now considered imprecise and outdated. Angular pregnancy is an ill‐defined clinical entity, traditionally referring to implantation of an embryo medial to the utero‐tubal junction, in the lateral angle of the uterine cavity [5]. This is in fact now considered a variation of normally implanted pregnancy, and it has been suggested that the term should be abandoned [4].

IEP is perhaps most frequently confused with cornual ectopic pregnancy. IEP differs from cornual implantation, which refers to pregnancies implanting in the upper and lateral uterine cavity rather than the tube, and may be ectopic (e.g., in the rudimentary noncommunicating horn of a unicornuate uterus) or nonectopic (e.g. in one horn of a bicorunuate uterus). Due to heterogeneity in clinical entities represented by cornual ectopic pregnancy, it has been suggested this terminology is also abandoned [6].

### 3.2. Ultrasound Characteristics

Accurate diagnosis of IEP can be challenging. Pelvic ultrasound, in particular, high‐resolution TVS, is considered the first‐line imaging modality for diagnosis of IEP [3].

ESHRE have recommended that standardised diagnostic criteria are developed to differentiate between different types of ectopic pregnancy, and propose that ectopic pregnancies should be broadly classified as uterine or extrauterine. Uterine ectopic pregnancies describe those which ‘breach the endometrial–myometrial junction and extend into the myometrium’ and should be further subclassified as partial or complete depending on the degree of myometrial involvement (4 p6). IEP traverses the boundaries of both uterine and tubal ectopic pregnancy. IEP has been highlighted as a uterine ectopic subtype particularly suitable for classification as partial or complete. However, reclassifying IEP as a type of tubal ectopic pregnancy has been proposed in order to encourage more conservative surgical approaches, and to reduce the use of uterine wedge resection. Challenges exist, however, due to a lack of defined anatomical barriers between the uterine cavity, fallopian tubes, and abdominal cavity [4].

Accurate use of terminology to describe the exact location of ectopic pregnancies on ultrasound is important to ensure timely diagnosis and to facilitate appropriate management. This is particularly important for IEP and uterine ectopic pregnancies with respect to the degree of myometrial involvement.

Differential diagnosis of IEP includes isthmic and intramural ectopic pregnancy. Isthmic pregnancies are defined by ESHRE as those located ‘close to the uterus but not surrounded by myometrium’ and are a type of tubal ectopic pregnancy [4]. Intramural pregnancies are described by ESHRE as those, which are located within the uterus but breach the endometrial–myometrial junction, in addition to invading the myometrium of the uterine corpus above the internal os. They are more common in the setting of previous myomectomy or classical caesarean incision. These do not, however, involve the interstitial portion of the fallopian tube, which helps to differentiate these from IEP [4]. Intramural pregnancy is a type of uterine ectopic pregnancy [4].

The RCOG describe three ultrasound criteria for diagnosis of IEP, including an empty uterine cavity; gestational sac located laterally in the interstitial portion of the fallopian tube, surrounded by less than 5 mm of myometrium in all imaging planes; and presence of the ‘interstitial line sign’. Further, where available, the use of three‐dimensional ultrasound may be helpful to further confirm two‐dimensional sonographic findings and in some scenarios, MRI may also aid diagnosis [7].

Our case highlights the complex diagnostic challenges, which exist in the presence of coexistent uterine pathology such as leiomyomata (fibroids). There is a paucity of literature regarding optimal diagnosis and management in complex cases in which there is concurrent existence of (interstitial) ectopic pregnancy and uterine fibroids [8].

Ectopic pregnancy in a fibroid uterus presents a higher degree of complexity, but in the presence of appropriate clinical expertise and surgical skill, optimal patient outcomes may be achieved.

### 3.3. Management

Expectant, medical, and surgical approaches are described in the management of IEP, with choice of treatment guided by the patient′s clinical picture and haemodynamic stability, sonographic features including size of the ectopic, and biochemical markers specifically *β*‐hCG, in addition to the surgical experience and skill of the clinical team [9].

Surgical treatment is indicated in certain instances, for example, when IEP is large, live, or in cases of rupture with haemoperitoneum. The optimal approach in surgical management of IEP is unclear [10]. Previous surgical approach was primarily laparotomy with uterine wedge resection; however, this is associated with high morbidity [11]. Advances in minimally invasive gynaecological surgery mean that laparoscopic approaches are favoured first line and have become the standard of care [3]. A number of laparoscopic surgical techniques are described in management of IEP, with cornuostomy (salpingostomy) and cornual (wedge) resection representing two of the most common approaches. Cornuostomy has been favoured over cornual resection in recent literature with suggested advantages including preservation of uterine architecture and maintaining fertility [3].

Cornual (wedge) resection carries a risk of future uterine rupture, due to interruption of the myometrium and possible extensive uterine scarring. The incidence of subsequent uterine rupture and dehiscence after wedge resection has been reported to be as high as 30%, which is significantly higher than that associated with myomectomy [9, 12].

The risk of intraoperative bleeding is higher for IEP than for other types of ectopic pregnancy. Approaches to minimising blood loss include the use of intramyometrial vasopressin to optimise haemostasis, as described in this case, and favoured by other reports in the literature [3] and national guidelines [9]. Use of vasopressin in this manner means that large and small IEP have become amenable to laparoscopic management. Alternative strategies include the use of a loop or purse‐string suture placed at the base of the ectopic pregnancy and temporary occlusion (clipping/clamping) of the uterine arteries [13].

Key steps in laparoscopic cornuostomy include incision of the uterine cornua, excision of the products of conception, cornual repair and confirmation of haemostasis. Finally, salpingectomy on the affected side has been recommended due to damage to the interstitial component of the tube rendering it useless [2]. In cases of rupture, control of bleeding can be challenging, particularly if aiming to preserve future fertility potential of the patient [10]. However, appropriate use of vasopressin, as highlighted in our case, may help in managing blood loss.

## 4. Conclusion

IEP although rare, is associated with unique diagnostic challenges and can be fatal if not identified and treated in a timely fashion. Management of IEP in the presence of uterine fibroids represents increased complexity in diagnosis and management, and there is a paucity of literature on the subject.

Our case supports the minority of existing literature demonstrating that laparoscopic cornuostomy and ipsilateral salpingectomy are safe and appropriate in experienced hands, even in the presence of ectopic pregnancy rupture with large haemoperitoneum.

## Funding

No funding was received for this manuscript.

## Disclosure

Dr. Orla Power affirms that this manuscript is an honest, accurate, and transparent account of the study being reported; that no important aspects of the study have been omitted; and that any discrepancies from the study as planned (and, if relevant, registered) have been explained. All authors have read and approved the final version of the manuscript. Dr Orla Power had full access to all of the data in this study and takes complete responsibility for the integrity of the data and the accuracy of the data analysis.

## Consent

Written informed consent was obtained from the patient for publication of this case report and accompanying images.

## Conflicts of Interest

The authors declare no conflicts of interest.

## Data Availability

Data sharing is not applicable to this article as no datasets were generated or analysed during the current study.

## References

[1] Nguyen T. H. , Interstitial Ectopic Pregnancy, Visual Encyclopedia of Ultrasound in Obstetrics and Gynecology. (2022) https://www.isuog.org/education/visuog/obstetrics/early-pregnancy/ectopic-pregnancy/uterine-ectopic-pregnancy/interstitial-ectopic-pregnancy.html.

[2] Whynott R. M. and Mikhail E. , Laparoscopic approach to Cornual Ectopic: A Step-by-Step Demonstration, Fertility and Sterility. (2019) 112, no. 2, 397–398, 10.1016/j.fertnstert.2019.04.030, 2-s2.0-85067977033, 31280953. 31280953

[3] Brincat M. , Bryant-Smith A. , and Holland T. K. , The Diagnosis and Management of Interstitial Ectopic Pregnancies: A Review, Gynecological Surgery. (2019) 16, no. 1, 10.1186/s10397-018-1054-4, 2-s2.0-85061077395.

[4] ESHRE Working Group on Ectopic Pregnancy , Kirk E. , Ankum P. , Jakab A. , Le Clef N. , Ludwin A. , Small R. , Tellum T. , Töyli M. , Van den Bosch T. , and Jurkovic D. , Terminology for Describing Normally Sited and Ectopic Pregnancies on Ultrasound: ESHRE Recommendations for Good Practice, Human Reproduction Open. (2020) 2020, no. 4, hoaa055, 10.1093/hropen/hoaa055, 33354626. 33354626 PMC7738750

[5] Jansen R. P. and Elliott P. M. , Angular Intrauterine Pregnancy, Obstetrics and Gynecology. (1981) 58, no. 2, 167–175. 7254728

[6] Baltarowich O. H. , The Term "Cornual Pregnancy" Should Be Abandoned, Journal of Ultrasound in Medicine. (2017) 36, no. 6, 1081–1087, 10.1002/jum.14207, 2-s2.0-85036577772, 28429456. 28429456

[7] Elson C. J. , Salim R. , Potdar N. , Chetty M. , Ross J. A. , and Kirk E. J. , Diagnosis and Management of Ectopic Pregnancy, BJOG: An International Journal of Obstetrics and Gynaecology. (2016) 123, no. 13, e15–e55, 10.1111/1471-0528.14189, 2-s2.0-85016925412. 27813249

[8] McDougall A. A. , Rouabhi S. , Magama Z. , and Odejinmi F. , Laparoscopic Myomectomy to Facilitate Laparoscopic Resection of a Bleeding Interstitial Ectopic Pregnancy, BMJ Case Reports. (2022) 15, no. 9, e250584, 10.1136/bcr-2022-250584, 36137642. PMC9511600 36137642

[9] Fee N. , Begley B. , McArdle A. , Milne S. , Freyne A. , and Armstrong F. , National Women and Infants Health Programme and The Institute of Obstetricians and Gynaecologists, 2024, The Diagnosis and Management of Ectopic Pregnancy, https://www.hse.ie/eng/about/who/acute-hospitals-division/woman-infants/clinical-guidelines/the-diagnosis-and-management-of-ectopic-pregnancy-2024-.pdf.

[10] Bhagavath B. and Lindheim S. R. , Surgical Management of Interstitial (cornual) Ectopic Pregnancy: Many Ways to Peel an Orange!, Fertility and Sterility. (2021) 115, no. 5, 1193–1194, 10.1016/j.fertnstert.2021.03.016, 33810847. 33810847

[11] Moon H. S. , Kim S. G. , Park G. S. , Choi J. K. , Koo J. S. , and Joo B. S. , Efficacy of Bleeding Control Using a Large Amount of Highly Diluted Vasopressin in Laparoscopic Treatment for Interstitial Pregnancy, American journal of Obstetrics and Gynecology. (2010) 203, no. 1, 30.e1–30.e6, 10.1016/j.ajog.2010.02.030, 2-s2.0-77953916863, 20451893. 20451893

[12] Liao C. Y. , Tse J. , Sung S. Y. , Chen S. H. , and Tsui W. H. , Cornual Wedge Resection for Interstitial Pregnancy and Postoperative Outcome, Australian & New Zealand Journal of Obstetrics & Gynaecology. (2017) 57, no. 3, 342–345, 10.1111/ajo.12497, 2-s2.0-84994726553, 27456318. 27456318

[13] Khan Z. and Lindheim S. R. , In Pursuit of Understanding Interstitial Pregnancies: A Rare Yet High-Risk Ectopic Pregnancy, Fertility and Sterility. (2019) 112, no. 2, 246–247, 10.1016/j.fertnstert.2019.05.027, 2-s2.0-85067959830, 31257000. 31257000

